# Exposure to Suicide in High Schools: Impact on Serious Suicidal Ideation/Behavior, Depression, Maladaptive Coping Strategies, and Attitudes toward Help-Seeking

**DOI:** 10.3390/ijerph15030455

**Published:** 2018-03-06

**Authors:** Madelyn S. Gould, Alison M. Lake, Marjorie Kleinman, Hanga Galfalvy, Saba Chowdhury, Alison Madnick

**Affiliations:** 1 Division of Child & Adolescent Psychiatry and Department of Epidemiology, Columbia University Medical Center and The New York State Psychiatric Institute, 1051 Riverside Drive, Unit 72, New York, NY 10032, USA; 2 Division of Child & Adolescent Psychiatry, The New York State Psychiatric Institute, 1051 Riverside Drive, Unit 72, New York, NY 10032, USA; alison.lake@nyspi.columbia.edu (A.M.L.); marjorie.kleinman@nyspi.columbia.edu (M.K.); saba.chowdhury@nyspi.columbia.edu (S.C.); alison.madnick@nyspi.columbia.edu (A.M.); 3 Departments of Psychiatry and Biostatistics, Columbia University Medical Center, 722 West 168 Street, New York, NY 10032, USA; hanga.galfalvy@nyspi.columbia.edu

**Keywords:** suicide, peers, exposure, negative life events, friendship, maladaptive coping attitudes, help-seeking attitudes

## Abstract

Adolescents’ exposure to a peer’s suicide has been found to be associated with, as well as to predict, suicidal ideation and behavior. Although postvention efforts tend to be school-based, little is known about the impact of a schoolmate’s suicide on the school’s student population overall. The present study seeks to determine whether there is excess psychological morbidity among students in a school where a schoolmate has died by suicide, and whether students’ attitudes about coping and help-seeking strategies are more or less problematic in such schools. Students in twelve high schools in Suffolk and Westchester counties in New York State—2865 students at six schools where a student had died by suicide within the past six months, and 2419 students at six schools where no suicide had occurred within the current students’ tenure—completed an assessment of their suicidal ideation and behavior, depressive symptoms, coping and help-seeking attitudes, stressful life events, and friendship with suicide decedent (if applicable). No excess morbidity (i.e., serious suicidal ideation/behavior and depression) was evident among the general student population after a schoolmate’s death by suicide; however, the risk of serious suicidal ideation/behavior was elevated among students at exposed schools who had concomitant negative life events. There was a significant relationship between friendship with the decedent and morbidity, in that students who were friends, but not close friends, of the decedents had the greatest odds of serious suicidal ideation/behavior. Overall, students in exposed schools had more adaptive attitudes toward help-seeking; but this was not true of the decedents’ friends or students with concomitant negative life events. The implications of the findings for postvention strategies are discussed.

## 1. Introduction

As suicide rates in the U.S. continue to rise [1], so does the concomitant number of individuals exposed to a suicide. With estimates of the number of individuals exposed to each suicide ranging from 10 [2] to 147 [3], between nearly 450,000 and over six million people would have been exposed to the 44,965 deaths by suicide in 2016, the year for which the latest data are available in the U.S. The number of individuals exposed to an adolescent’s suicide would range from approximately 24,000 to 360,000 given the 2439 13–19 year olds who died by suicide in 2016 [4]. Understanding the impact of this exposure is of critical import in developing effective postvention strategies—“activities which occur after a suicidal event” to reduce “the psychological sequelae of a suicidal death in the survivor-victim” [5] (pp. 21–22).

The effects of a peer’s suicide on adolescents’ mental health problems and suicide risk has been the focus of myriad studies [6,7,8,9,10,11]. The majority of cross-sectional analyses examining adolescents’ exposure to a peer’s suicidal behavior have found a significant association with adolescent suicidal ideation and attempts [6,9,10,11]. Consistent findings have emerged from several longitudinal studies using data from the National Longitudinal Survey of Adolescent Health (ADD Health) [7,8,9,11]. Similarly, a longitudinal study employing the Canadian National Longitudinal Survey of Children and Youth [12] found that exposure to a schoolmate’s attempted or completed suicide predicted suicidal ideation and attempts among youth two years after exposure. Effects of suicide exposure on suicidality outcomes were not modified by previous social support, depression, or anxiety, or previous ideation or attempts. However, students who had experienced stressful life events appeared more affected than those without such life events. While closeness to a suicide decedent appears to consistently enhance the impact of the exposure among adults [13,14], the findings among adolescents are inconsistent. Some studies of adolescents [12,15] did not find closest friends of a suicide decedent to be at heightened risk of suicidality compared with acquaintances, while others [10] reported that a close relationship with a suicide attempter or completer increased risks for suicidal behaviors.

The studies that have examined the impact of a peer’s suicide to date were not designed to determine the extent to which excess psychological morbidity, specifically serious suicidal ideation/behavior and depression, exists among students in a school where a schoolmate has died by suicide. It is also currently unknown whether attitudes about coping strategies and help-seeking are more or less problematic in a school where a schoolmate has died by suicide. The aim of the current study is to address these questions. An examination of these questions requires a case-control study comparing students in schools with and without a suicide. The studies examining the impact of a peer’s suicide have instead usually employed general population surveys of youth [9,12], and the one other study that employed a case-control sample of schools [10] did not attempt to address these issues. A question also addressed by the present case-control study is whether schoolmates’ suicide risk, depression and attitudes differ as a function of their concomitant undesirable life events or their level of friendship with the suicide decedent. An examination of these questions can inform the design of school-based suicide postvention programs, which have rarely been shaped by empirical evidence [16].

## 2. Materials and Methods

### 2.1. Sample

The present study is a case-control study in which the cases were students within high schools in Suffolk and Westchester counties in New York State where a schoolmate had died by suicide (“exposed” schools). For a school to be eligible, the verdict of the suicide had to be made by the county medical examiner’s office within a few months of the death enabling us to recruit the school and conduct the assessment within six months of the death. The suicide decedent had to be enrolled in school at the time of death, and the cause of death known to the school, so that a targeted school would be the type of school at which a postvention program is most apt to be implemented. Furthermore, no other student suicide could have occurred within four years of the index student suicide (a time period equal to students’ tenure in high school). A total of 14 schools met our eligibility criteria, of which six agreed to participate. To be eligible as one of the six “non-exposed” control schools, no completed suicide by a student or school employee could have occurred within four years of the assessment; the school was within a school district non-contiguous to an exposed school, but within the same or adjacent county as an exposed school; and was of comparable socioeconomic status (e.g., comparable percentage of free or reduced-price lunches in schools) to an exposed school. All of the high schools were in suburban communities surrounding New York City. The schools were moderately socioeconomically diverse; the percentage of free or reduced-price lunches ranged from <1% to 12.5%.

We assessed 2865/4285 enrolled students in the exposed schools (66.9% participation rate) and 2419/3815 in the non-exposed (63.4% participation rate) commencing in the fall of 1998 and ending in the spring of 2001. The overall participation rate was 65.2%. The ethnic distribution of the participating sample in the exposed schools was 87.0% White, 2.3% African American, 4.1% Hispanic, 3.1% Asian, and 3.6% other; approximately 58% of the students were male. The ethnic distribution of the participating sample in the non-exposed schools was 77.7% White, 5.5% African American, 7.4% Hispanic, 3.8% Asian, and 5.7% other; approximately 58% of the students were male. The mean age of participating students was 15.5 years in both exposed and non-exposed schools and 92% of the students were between ages 14 and 17. There were no significant differences between participants and non-participants in gender, grade level, and ethnicity. Furthermore, there were no significant differences in the gender or age distributions between participants in the exposed and non-exposed schools; however, there were slightly more White participants in the exposed schools (*p* < 0.001).

### 2.2. Procedure

**Figure 1:**
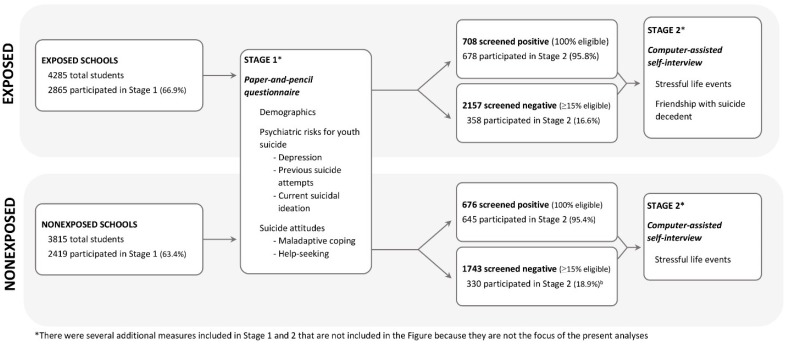
Two-Stage Study Procedure and the Flow of Participants.

A two-stage (two day) assessment process was used in this study (see Figure 1). During stage 1, all participating students completed a paper-and-pencil questionnaire that screened for psychiatric risks for youth suicide [17] (described below in the Measures section). All students who screened positive and approximately 15% of those who screened negative (minimum of 50 students per school) were invited to participate in stage 2, which took place within two days of stage 1. Students screened positive if they met any of the following criteria: (1) responded either “I would like to kill myself” or “I would kill myself if I had the chance” on the suicide item on the Beck Depression Inventory (BDI-IA) [18]; (2) thought about suicide most days or every day; (3) answered yes to any of the following questions: “Have you thought seriously about killing yourself?” “Are you still thinking about killing yourself?” “Have you made a specific plan about how you would kill yourself?” “Have you tried to kill yourself in the past four weeks?” “Have you ever tried to kill yourself, before the past four weeks?” or “Suicide is a good (or the only) solution to problems”; (4) scored greater than or equal to 13 on the BDI-IA; (5) reported significant drug use or impairment from drugs (e.g., problems in school adjustment, behavior, health, peer relations) based on the Drug Use Screening Inventory [19]; (6) indicated a desire to see a health professional in response to a question at the end of stage 1; or (7) did not respond to questions about suicide (if they also scored above or equal to 11 on BDI). A total of 708 students screened positive in the exposed schools, of whom 678 (95.8% participation rate) participated in stage 2. A total of 676 students screened positive in the non-exposed schools, of whom 645 (95.4% participation rate) participated in stage 2. Within two days of the completion of stage 1, the responses were scored via an optical scanner. This enabled stage 2 assessments to start within two days of stage 1.

During stage 2, students completed a computer-assisted self-interview. As a safety procedure, results of the computerized interviews were immediately reviewed by a study clinician (child psychiatrist, psychologist, or social worker). Clinical interviews were conducted with students who scored positively on any of the suicide items during stage 1; indicated a desire to see a health professional in response to a question at the end of stage 1; did not respond to questions about suicide (if they also scored above or equal to 11 on BDI); or indicated serious suicidal ideation or a history of suicidal behaviors during stage 2. For students in need of a referral, follow ups were arranged by a research team case manager (a certified social worker) who worked with the parents and school guidance personnel under the supervision of a qualified psychiatrist.

To recruit students for the study, the schools utilized an opt-out procedure for parents and active written assent for the students. The schools sent out two mailings to these students’ homes, which included information about the assessments, a response form, and a stamped return envelope. These mailings were sent out four and two weeks prior to stage 1, which gave parents the opportunity to remove their child from participation. On the day of stage 1, schools obtained written assent from the students. The study’s procedures abided by the Family Educational Rights and Privacy Act and the Protection of Pupil Rights Amendment and were approved by the Institutional Review Board of the New York State Psychiatric Institute/Columbia University Department of Psychiatry (#4654R).

### 2.3. Measures

The timeframe of the stages 1 and 2 assessments was the past four weeks (including the day of the assessment), unless otherwise noted. Stage 1 included measures of demographics (age, gender, race/ethnicity); depression; suicidal ideation and behavior; and suicide attitudes. Stage 2 included measures of stressful life events and, for exposed schools only, an assessment of the degree of friendship with the suicide decedent. There were several additional measures included in stages 1 and 2 that are not described below because they are not the focus of the present analyses.

*Depression*. The Beck Depression Inventory (BDI-IA) [18] was utilized to assess adolescent depression. Specifically, the BDI items evaluated cognitive, behavioral, affective, and somatic factors of depression. All of the original scale items except for loss of libido were included. For each question response, the scores ranged from 0 (no symptoms present) to 3 (symptoms are severe); the maximum total score was 60. In order to dichotomize scores for this study, Beck’s recommended cutoff point of 16 was used. In Beck’s study, this cutoff was suggested to detect depression in normal populations. Additionally, in Strober’s study, this cutoff correctly classified 81% of the study sample of adolescent psychiatric patients with major depressive disorder [20].

*Suicidal Ideation/Behavior*. Students were asked eight questions about suicidal ideation and six questions about suicide attempts. One ideation question was obtained from the BDI, while the rest of the yes/no questions were taken from a depression module of the Diagnostic Interview Schedule for Children Version 4 [21] and an earlier suicide screening assessment [22]. All of the questions chosen for this study have previously demonstrated good construct validity by their significant association with psychopathology [22,23]. We considered ideation to be serious if thoughts were rated as occurring most days or every day or if the respondent was still thinking about suicide on the day of the assessment, had made a specific plan, responded affirmatively to “Have you thought seriously about killing yourself?” or responded with either of the two most serious response options on the BDI suicide item: “I would like to kill myself” or “I would kill myself if I had the chance.” A student was categorized as currently having serious suicidal ideation/behavior if he or she met these criteria or had made an attempt (regardless of injury or medical attention) within the 4 weeks prior to the assessment.

*Suicide Attitudes*. The attitude assessment consisted of 18 items. This assessment was developed for a prior study [24], and the items were previously shown to have good reliability and validity. Students were asked whether they agreed with ten statements, and in response to the following question, “What should you do if a friend tells you he/she is thinking about killing himself/herself?”, students were given eight yes/no response options. An exploratory factor analysis was performed to ascertain the number of factors underlying the 18 items [25]. The factor analysis yielded a three-factor model, of which the first two factors, maladaptive coping strategies (MCS; seven items) and help-seeking strategies (HSS; five items), were found to be reliable (Cronbach’s alpha coefficient = 0.54, 0.60, respectively). The third factor was not conceptually related to coping strategies and is not included in the present paper. MCS reflected dysfunctional attitudes that would appear to support isolative behaviors and a tendency towards self-sufficiency, rather than encourage help-seeking for suicide and depression. For example, youth endorsing the attitudes on this factor would think that people should be able to handle their own problems without outside help, that it is a good idea to keep feelings of depression to yourself or alleviate these problems with drugs and alcohol, and rather than getting help for a suicidal friend, these youth would keep the confidence a secret, or not take it seriously. In contrast, the HSS factor consisted of healthy coping strategies that involved actively seeking advice and appropriate services from others—most typically, an adult/authority figure—for a suicidal person. The correlation between the two factors was −0.30. A total score on each factor was calculated by summing the student’s positive responses to each of the component items (range for MCS = 0 to 7; range for HSS = 0 to 5). The attitude questions were added to stage 1 after two of the exposed schools were assessed; therefore, they are available for four exposed schools and six non-exposed schools.

*Stressful Life Events*. The Coddington Life Events Scale (CLES) is a 50-item self-administered instrument designed to assess a range of events recently experienced by children and adolescents [26]. The CLES has been the focus of over 50 research studies and has established test-retest reliability and content, concurrent, predictive and discriminant validity. The Undesirable Life Events subscale was employed in the current analyses. This scale includes 25 negative life events varying in severity and the amount of social readjustment required by the youth. An example of an extreme event is the death of a parent (weight of 108). Examples of other negative events included breaking up with a girlfriend or boyfriend (weight of 39), failing a grade in school (weight of 47), and hospitalization for illness or injury (weight of 50). Following CLES’ instructions, the frequency of individual negative life events was multiplied by established weights, reflecting an event’s severity and impact on an individual’s life, to produce a total negative Life Change Unit (LCU) score. The event ‘death of a close friend’ was excluded from the calculation of the total negative LCU score in light of the inherent difference between exposed and non-exposed schools. The possible range of total negative LCU scores is 0 to 1209. The CLES’ time frame was modified from its assessment of “0–3 months ago” to the month prior to the assessment, to keep the time frame consistent across the various study measures.

*Friendship with Suicide Decedent*. Developed for the present study, the Friendship scale was comprised of the following four items, each scored on a four-point Likert scale:

(1) How well did you know him or her? (0 = not at all; 1 = acquaintances; 2 = friends, but not close friends; 3 = close friends, current boyfriend or girlfriend, ex-boyfriend or girlfriend);
(2) How often did you see him or her at school? This was a composite (highest response code) of two questions: “How often did you see him or her in class?” and “How often did you see him or her at school, other than in class?” (0 = never; 1 = less than once a week; 2 = once to four times a week; 3 = daily);
(3) How often did you see him after school or talk on the phone? This was a composite of “How often did you see him or her after school or on weekends?” and “How often did you talk to him or her on the phone?” (0 = never; 1 = less than once a week; 2 = once to four times a week; 3 = daily);
(4) To what extent did you confide in each other? This was a composite of “Was he or she somebody in whom you could confide?” and “Were you someone in whom he or she confided?” (0 = Not at all; 2 = somewhat; 3 = very much so).

A student’s total score (ranging from 0 to 12) was calculated by adding his/her scores on each of the four component items. The Cronbach’s alpha for the total Friendship scale score was 0.81.

### 2.4. Analytic Strategy

To test for the associations between exposure to a schoolmate’s suicide and the dichotomous outcomes (serious suicide ideation/behavior; depression; individual coping and help-seeking items) and continuous outcomes (coping and help-seeking attitude scales), we first modeled each of the outcome variables separately as a function of the schools’ exposure group status, using mixed effects logistic and linear regression models, respectively. Random intercept effects were included for each school nested into the exposure group to account for variability shared by students in the same school. The fixed effect predictor was the schools’ exposure group status. The analyses examining the attitude outcomes employed four exposed schools rather than six, and six non-exposed schools because, as previously noted, these attitudinal questions were added to stage 1 after two exposed schools had been assessed. Next, mixed effects multiple logistic and linear regression models were tested—with school exposure group, negative life events and an interaction term between negative life events and school exposure status as the predictors—to determine the significance of differences in outcomes between the students in the groups of exposed and non-exposed schools, the impact of negative life events, and whether life events modified the impact of exposure. Lastly, the impact of friendship with the decedent (friendship scale and individual friendship characteristics) on the outcomes was evaluated within the group of exposed schools by employing a series of mixed effects logistic and linear regression models. All analyses were adjusted for gender, age and ethnicity. The analyses that included variables from stage 2 (negative life events and friendship with the decedent) were weighted to adjust for unequal selection probabilities of stage 1 positives and negatives for inclusion in stage 2. Statistical analyses were conducted using SAS version 9.4 (SAS Institute Inc., Cary, NC, USA) [27] and IBM SPSS version 23 (IBM Corp., Armonk, NY, USA) [28].

## 3. Results

### 3.1. Association of Exposure with Psychological Morbidity and Coping/Help-Seeking Attitudes

The relationships between students’ exposure to a schoolmate’s suicide and their own suicidal ideation/behavior, depression and attitudes about their coping strategies and help-seeking are presented in Table 1. 

There was no significant excess in psychological morbidity, as reflected by serious suicidal ideation/behavior and depression, among the general student population after a death by suicide of a schoolmate. The mean score on the maladaptive coping scale was significantly lower among students in the exposed schools than non-exposed schools (*p* < 0.05) and conversely, there was a trend for the help-seeking scale scores to be higher among exposed students (*p* = 0.062). Specifically, the exposed students had significantly lower odds than non-exposed students of agreeing with the statements, “people should be able to handle their own problems without outside help” (*p* < 0.01) and “drugs and alcohol are a good way to help someone stop feeling depressed” (*p* < 0.05). Moreover, in response to a friend telling them that they were suicidal, exposed students had significantly higher odds of reporting that they would talk to an adult about their friend (*p* < 0.01), and there was a trend for them to be more likely to tell their friend to talk to his/her parents (*p* = 0.062). Conversely, there was a trend for exposed students to have lower odds of talking to a friend without getting anyone else’s help (*p* = 0.094) or not taking their friend’s suicidal disclosure seriously (*p* = 0.069).

### 3.2. Association of Negative Life Events with Psychological Morbidity and Coping/Help-Seeking Attitudes

The results of the analyses examining the association of negative life events with serious suicidal ideation/behavior, depression and the coping/help-seeking attitudes, and the interactions between negative life events and exposure are presented in Table 2. Students reporting more negative life events had significantly greater odds of reporting serious suicidal ideation/behavior (*p* < 0.0001), depression (*p* < 0.0001) and endorsing attitudes that reflect maladaptive coping strategies (*p* < 0.0001). They had lower odds of endorsing adaptive help-seeking attitudes (*p* = 0.001). The main effect of exposure on all the outcomes was not statistically significant; however, while the interactions between exposure and negative life events were not significant with regard to depression or the attitude scales, there was a significant interaction between exposure status and negative life events with regard to their impact on serious suicidal ideation/behavior (*p* = 0.007). Students with recent negative life events who attended schools where a schoolmate had recently died by suicide had significantly greater odds of serious suicidal ideation/behavior than students with recent negative life events in non-exposed schools.

### 3.3. Association of Friendship with the Suicide Decedent with Psychological Morbidity and Coping/Help-Seeking Attitudes

The percentages of students in the exposed schools reporting serious suicidal ideation/behavior and depression, and the means and standard deviations on the coping/help-seeking attitude scales across different levels and types of friendship with the decedent are presented in Table 3. The results of the regression analyses examining the significance of the relationships between the friendship variables and each of the four main outcomes are presented in Table 4.

Over half of the students in the exposed schools (1568/2867, 54.7%) described themselves as not knowing the suicide decedent at all; 27.6% (790/2867) reported they were the decedent’s acquaintance; 14.3% (410/2867) reported they were friends, but not close friends; and 3.4% (97/2867) said they were close friends (weighted Ns and percentages, see Table 3). Approximately one third of students (32.7%) reported that they never saw the decedent in school (21.6% less than once a week; 15.0% once to four times a week; 30.1% daily). Approximately three fourths (75.6%) reported that they never saw or talked to the decedent outside of school (15.2% less than once a week; 6.3% once to four times a week; 2.8% daily). Few students reported that they and the decedents were confidants (83.8% not at all; 10.9% somewhat; 3.5% very much so).

Overall, within the exposed group of schools, students with higher scores on the total Friendship scale had significantly greater odds of serious suicidal ideation/behavior (*p* < 0.0001) and depression (*p* < 0.0001) and higher mean scores on the maladaptive coping strategies scale (*p* < 0.05). Friendship with the decedent was not significantly associated with adaptive attitudes toward help-seeking (*p* = 0.399). The highest rates of serious suicidal ideation/behavior were not reported by decedents’ closest friends, as assessed by the total friendship score, how well the decedent was known to the student and the extent to which they confided in each other (Table 3 and Table 4). Only friends who were not close to the decedent had significantly increased odds of serious suicidal ideation/behavior compared to the students who did not know the decedent at all (*p* < 0.01). Similarly, those who reported that they and the decedent confided in each other a lot did not have increased odds of serious suicidal ideation/behavior compared to those who did not confide with the decedent (*p* = 0.643); rather, only those who reported that they and the decedent confided in each other somewhat had increased odds of serious suicidal ideation/behavior (*p* < 0.0001). A different pattern emerged for depression; any students who knew or were confidants of the decedent had greater odds of depression than students who did not know or confide with the decedent. Only acquaintances of the decedent had significantly higher scores on the maladaptive coping strategies scale score (*p* < 0.001).

## 4. Discussion

The primary aims of the study were to determine the extent to which excess psychological morbidity exists among students in schools where a schoolmate has recently died by suicide and to examine whether attitudes about coping strategies and help-seeking are more or less problematic among these exposed students. Our findings indicated that there was no excess of serious psychological morbidity, as reflected by rates of serious suicidal ideation/behavior and depression, among the general population of students attending schools where a schoolmate had died by suicide within the past six months. However, among students with recent negative life events, exposure to a schoolmate’s suicide significantly increased their risk of serious suicidal ideation/behavior compared to comparable students in non-exposed schools. Friendship with the decedent was associated with psychological morbidity; however, it was not the closest friends of the decedents who were at increased risk of serious suicidal ideation/behavior; rather it was less close friends who had the highest rates of serious suicidal ideation/behavior. In contrast, elevated rates of depression were evident among students who knew the decedent at any level—acquaintances, friends, close friends—compared to those who did not know the decedent at all. Exposure to a suicide in a school was associated with a reduction in dysfunctional attitudes related to isolation and self-sufficiency, and conversely, with an increase in adaptive help-seeking attitudes among the general student population. However, this was not true for friends of the decedent or those with more negative life events.

The impact of exposure to a suicide death is not monolithic. A “continuum of survivorship” has been postulated whereby most people who are exposed to suicide will not be affected or experience either short-term or long-term dysfunction [29]. Our results are consistent with this model. Over half of the exposed students attending schools where a schoolmate recently died by suicide reported that they did not know the decedent at all. These individuals appeared not to be affected by the death in terms of their rates of serious suicidal ideation/behavior and depression, which resembled the rates among students in the non-exposed schools. According to the continuum of survivorship model, those who are affected by a suicide death are likely to include close friends, but are not limited to close friends. Among a sample of exposed adults, increased “perception of closeness” to the decedent increased the odds of psychological sequelae, including depression, anxiety and posttraumatic stress disorder [13]. Similarly, among students exposed to a completed suicide, close friends had a higher risk for internalizing problems [10]. Our results are partially consistent with these findings. In our sample of exposed students, friendship with the decedent increased the odds of depression, with nearly one quarter of close friends scoring in the clinical range on the depression scale. However, the relationship between friendship and serious suicidal ideation/behavior was more complicated. The decedent’s close friends were not at increased odds of serious suicidal ideation/behavior; rather it was less close friends who were at highest risk.

Experiencing negative stressful life events also appears to moderate the effect of exposure. The increased risk of suicidal behavior manifested by youth experiencing negative stressful life events has long been established [30,31]. However, the synergistic effect of suicide exposure and negative stressful life events on increasing suicide ideation and behavior has rarely been studied. An earlier longitudinal study [12] found that exposure to a schoolmate’s suicide was predictive of suicidal ideation and behavior only for those students with previous stressful life events, but not for exposed youth with no previous stressful life events. The current result of a significant effect of the interaction between concomitant negative stressful life events and recent exposure to a schoolmate’s suicide on increasing the risk of serious suicidal ideation/behavior is consistent with the earlier finding.

There has been a suggestion that exposure to suicide can have an inhibitory effect on risk for suicidal behavior [32]. Our findings are consistent with this idea in that whereas the decedents’ closest friends had the highest rates of depression, they did not have the highest rates of serious suicidal ideation/behavior. Furthermore, our finding that exposure to a schoolmate’s suicide reduced endorsement of maladaptive coping strategies and enhanced help-seeking attitudes among the general population of students suggests one mechanism by which exposure may exert an inhibitory effect. The exposure to the suicide death may have highlighted the need for students to become more vigilant about suicidal peers and actively seek help for them.

Our findings can be used to address the competing hypotheses regarding mechanisms by which exposure exerts its influence. These hypotheses include contagion, whereby there is a direct causal effect of exposure [33], and assortative relating, whereby individuals at high risk of suicidal behavior are more likely to be friends with other high risk individuals, and this shared risk explains the effect of exposure [11,34]. Our findings do not appear to be consistent with assortative relating because this mechanism would have predicted that friends closest to the suicide decedents would have been the most suicidal, but that was not the case. Recent research employing the ADDhealth longitudinal surveys [11] also highlighted that assortative relating was not sufficient to explain the increased risk after exposure to peer suicidal behavior; even after controlling for a wide range of potential variables potentially “involved in assortative selection of peers” [11] (p. 220), being exposed to a peer’s suicidal behavior still increased the risk of suicidal behavior. Our lack of a significant main effect of exposure on serious suicidal ideation/behavior and depression would also appear not to be consistent with contagion; however, a contagion hypothesis does not propose that the effect of exposure is uniform in a population; rather, forces within the individual (“host susceptibility”), as well as the environment influence the impact of exposure on health outcomes [35,36]. Moreover, it has been suggested that in suicide contagion, “a ‘social multiplier’ may amplify the effects” of other suicidogenic factors [37] (p. 233). In our findings, exposure appears to amplify the effects of negative life events on serious suicidal ideation/behavior. As such, this finding is consistent with a process of suicide contagion.

It is worth exploring the possibility that some of the effects we found could be partially explained by the impact of the exposed schools’ crisis responses to the suicide deaths, rather than by the suicide deaths themselves. Postvention programs were not as extensive at the time of the study as they currently are; nevertheless, each of the exposed schools implemented a crisis response that included sharing basic facts about the death and suicide risk with students, and expanding students’ access to guidance personnel, particularly for students thought to be at risk by virtue of their being previously known to guidance personnel. It would be surprising for such minimal intervention to have a highly protective effect on the student population; nonetheless, it is conceivable that the exposed schools’ postvention efforts could have contributed to the overall lack of excess morbidity among students at those schools, as well as to the overall improvements in students’ coping and help-seeking attitudes. In the event that close friends of the decedent may have been a focus of postvention efforts in and outside of school, these interventions could also have contributed to the lack of excess serious suicidal ideation/behavior we found among this group. Whether or not the postvention activities implemented by the exposed schools contributed to these effects, our findings suggest that future postvention programs should direct increased attention to students who are less close friends of the decedent, and to students with concomitant negative life events, including those who may not previously have manifested noticeable symptomatology, and who therefore may not previously have come to the attention of guidance personnel. Whatever effects the exposed schools’ minimal postvention programs may have had on the student population at large, or on the subgroups targeted by the programs, our findings indicate that they did not adequately protect these two groups of vulnerable students.

The present study has several strengths. First, the case-control design, comparing students in schools with and without a suicide, enabled us to examine whether there was an excess of psychological morbidity in exposed schools. This is the first study to our knowledge to address this question. Second, the exposed schools reflected real-world settings that exemplify the situations where postvention programs are most apt to be implemented. Third, our establishment of relationships with the medical examiners’ offices in the study counties permitted weekly telephone contacts that yielded the identification of youth suicides within a relatively short period of time after their deaths. This allowed the assessment of their schoolmates within a time period that could inform postvention strategies. Fourth, the large sample yielded ample statistical power to detect interactions between exposure and comorbid vulnerabilities, such as students’ experience of negative life events.

The study also has important limitations. One limitation was the employment of suburban schools with predominantly white populations, so that the results cannot be generalized to urban and more ethnically diverse settings. Design considerations dictated the project’s implementation in suburban counties surrounding NYC, rather than in NYC (which has a more ethnically and socioeconomically diverse population) because lengthy delays in the adjudication of suicides in the NYC Medical Examiner’s office precluded the timely implementation of the assessment protocol within the city. Furthermore, most youth suicides are white in the suburban counties surrounding NYC; consequently, the exposed schools in our project were comprised of a largely white population. A second limitation was the low student participation rate, which was nonetheless comparable to that achieved by other suicide screening protocols [22,38,39]. Reasons for low student participation in school-based suicide screening protocols include parents’ and students’ concerns about the confidentiality of the screening results, and their reluctance to accept help even if a problem were to be identified, diminishing the relevance of a screening protocol [39]. Given the similar participation rates in the exposed and non-exposed schools, it appears unlikely that this affected our findings. There were no significant differences between the participants and non-participants in gender, grade level, and ethnicity. However, it is unknown whether participants may have differed from nonparticipants on other study measures. A third limitation was the low school participation rate. The main reasons for a school’s refusal to participate included the administrator’s perceived inconvenience of implementing a school-wide screen, and concerns about the school’s liability after identifying at-risk students. This is consistent with the widespread finding that school-wide suicide screening programs have been rated by high school principals and other school personnel as significantly less acceptable than curriculum-based and staff in-service programs [39,40,41,42]. A fourth limitation is that the data was collected between 1997 and 2001; therefore, a question could be raised as to the relevance of the findings. However, the existing research on school-based postvention programs is very limited so our empirical data to inform current practices is still timely. A fifth limitation is that all the exposed schools implemented similar postvention strategies so there was insufficient variability to examine differential effects of various postvention activities or to examine the overall impact of postvention strategies. Because the exposed schools each engaged in some postvention activity, we are unable definitively to distinguish the effects of the student suicide from the effects of the schools’ crisis response to the suicide. However, because the postvention activities that were typically implemented at that time were minimal, we are confident that our study design enabled us to examine the impact of exposure to suicide absent extensive postvention programming. As noted above, our findings identify two groups of students (less close friends of the decedent, and those with concomitant negative life events) in particular need of additional help. Sixth, because data on the exposed students’ pre-existing suicidal ideation and behavior, depression and attitudes were not available, we were unable to assess change, per se, as a function of exposure. We can merely assume that the students in the non-exposed schools reflected the pre-existing characteristics of the students in the exposed schools, and that differences between the groups estimated changes due to exposure. To make this more likely, we attempted to enhance the similarity of students in the exposed and non-exposed schools by selecting non-exposed schools that were comparable to exposed schools on socioeconomic indicators. Seventh, there was no longitudinal follow-up of the students, so it is unknown how symptoms seen in the first six months after a suicide may have evolved over time. Lastly, our focus was on the impact of a student suicide on the student population of a school; as such, we did not assess students’ exposure to any other suicide. Such exposure would likely constitute an additional risk factor we have not accounted for. However, there is no reason to believe that exposure to suicide outside of school would not be equally distributed across students from exposed and non-exposed schools, making it unlikely to impact our outcomes.

## 5. Conclusions

The current findings have significant implications for school-based suicide postvention programs. Postvention guidelines developed for schools over the last 20 years include the identification and support of high-risk students as a common element [16]. Those closest to the deceased students are often nominated to be at heightened risk after a schoolmate’s suicide [43]. However, the current findings indicate that acquaintances and friends less close to the decedent are at elevated risk of suicidal ideation and behavior, potentially even more so than friends closest to the deceased students, who may tend to receive more support. Existing postvention programs also do not acknowledge students’ negative life events as increasing risk for suicidal ideation and behavior; yet, our current findings indicate that the students who have experienced concomitant negative life events will need special attention after a schoolmate’s death by suicide, even if they have not previously manifested noticeable emotional or behavioral symptoms. Lastly, our findings highlight an opportunity for postvention programs to reinforce positive changes in adolescents’ attitudes about coping strategies and help-seeking among the general population of students after a schoolmate’s suicide, and to counteract the maladaptive coping and help-seeking attitudes that at-risk students often have [25]. Sources of Strength is a suicide prevention program that is based on the premise that the influence of peer leaders will be effective at transforming students’ attitudes and that the school climate can be positively impacted by peer influence and positive messaging [44]. Postvention programs can be shaped in a similar fashion. While the current study has provided valuable empirical data to shape postvention efforts, additional empirical data is needed to continue to better plan and evaluate school-based postvention programs; otherwise, they are liable not to adequately address the needs of the survivors and may not use resources effectively.

## Figures and Tables

**Table 1:** Associations of Exposure to Schoolmate’s Suicide in School with Suicidal Ideation/Behavior, Depression, Maladaptive coping strategies and Help-seeking strategies.

Outcome Measures	Exposed Schools (*n* = 2865) *n* %	Non-Exposed Schools (*n* = 2419) *n* %	OR or B (95% CI)	*p*
**Serious suicidal ideation/Behavior**	199, 6.9%	150, 6.2%	1.18 (0.85–1.64)	0.299
**Depression** (>BDI cutoff)	279, 9.8%	232, 9.7%	1.02 (0.69–1.50)	0.930
**Maladaptive Coping Scale ^a^** mean (SD)	0.699 (1.06)	0.863 (1.17)	−0.156 (−0.316–−0.01)	0.044
*-People should be able to handle their own problems without outside help*.	248, 12.6%	411, 17.0%	0.71 (0.57–0.87)	0.005
*-Drugs and alcohol are a good way to help someone stop feeling depressed.*	162, 8.2%	258, 10.7%	0.72 (0.55–0.94)	0.022
*-If you are depressed, it is a good idea to keep these feelings to yourself.*	107, 5.4%	183, 7.6%	0.77 (0.54–1.11)	0.137
*-Suicide is a possible solution to problems* ^b^	217/1916, 11.3%	299/2343, 12.8%	0.93 (0.70–1.23)	0.549
*-I would talk to my friend without getting anyone else’s help.*	410, 20.8%	581, 24.0%	0.82 (0.64–1.05)	0.094
*-I wouldn’t take it seriously.*	94, 4.8%	167, 6.9%	0.75 (0.54–1.03)	0.069
*-I would keep it a secret.*	135, 6.9%	187, 7.7%	0.93 (0.63–1.36)	0.659
**Help-seeking Scale ^a^** mean (SD)	3.01 (1.41)	2.83 (1.52)	0.175 (−0.01–0.36)	0.062
*-Tell my friend to see a mental health professional*	1405, 71.4%	1703, 70.4%	1.02 (0.84–1.25)	0.796
*-Tell my friend to call a hotline.*	880, 44.7%	1069, 44.2%	1.09 (0.69–1.71)	0.687
*-Tell my friend to talk to his/her parents.*	1354, 68.8%	1520, 62.8%	1.26 (0.99–1.61)	0.062
*-Talk to an adult about my friend.*	1457, 74.0%	1618, 66.9%	1.45 (1.13–1.85)	0.008
*-Get advice from another friend.*	830, 42.2%	944, 39.0%	1.13 (0.82–1.57)	0.403

^a^ *n* equals 1969 because the attitudes were assessed in only four of the six exposed schools; ^b^ Ns equal 1916 and 2343 for the exposed and non-exposed schools, respectively, due to missing values on this item.

**Table 2:** Mixed effect regression analyses of serious suicidal ideation/behavior, depression, and attitudes about coping and help-seeking strategies by school exposure and negative life events.

Main Effects and Interaction Terms	Serious Suicidal Ideation/Behavior		Depression		Maladaptive Coping Strategies		Adaptive Attitudes toward Help-Seeking	
	OR (95% CI)	*p*	OR (95% CI)	*p*	B (95% CI)	*p*	B (95% CI)	*p*
**School exposure status**	0.818 (0.522–1.283)	0.344	0.905 (0.560–1.462)	0.653	−0.147 (−0.388–0.094)	0.213	0.044 (−0.271–0.360)	0.767
**Negative Life Events**	1.005 (1.004–1.006)	<0.0001	1.005 (1.004–1.006)	<0.0001	0.002 (0.001–0.003)	<0.0001	–0.001 (−0.002–−0.001)	0.001
**Interaction: School exposure by negative events**	1.002 (1.001–1.004)	0.007	1.001 (0.999–1.003)	0.292	0.001 (−0.000–0.002)	0.272	0.001 (−0.001–0.002)	0.342

**Table 3:** Degree of friendship and serious suicidal ideation/behavior, depression, and attitudes about coping and help-seeking strategies in exposed schools.

Friendship Items	Serious Suicidal Ideation/Behavior ^+^		Depression ^+^		Maladaptive Coping Strategies ^++^		Adaptive Attitudes toward Help-Seeking ^++^	
	*n* ^a^	% ^a^	*n* ^a^	% ^a^	Mean ^b^	SD ^b^	Mean ^b^	SD ^b^
***Friendship scale score ^+^***								
0 (*n* = 867)	56	6.42	65	7.58	0.51	0.95	2.92	1.49
1–4 (*n* = 1306)	75	5.78	104	8.01	0.86	1.07	3.00	1.37
5–8 (*n* = 478)	58	12.08	69	14.49	0.96	1.13	2.86	1.46
9–12 (*n* = 192)	21	10.88	33	17.99	0.79	1.15	3.14	1.30
***How well did you know him/her? ^+^***								
Not at all (*n* = 1568)	100	6.36	118	7.59	0.63	0.97	2.93	1.43
Acquaintances (*n* = 790)	61	7.67	85	10.80	1.01	1.19	3.08	1.37
Friends, but not close friends (*n* = 410)	43	10.51	51	12.51	0.76	1.03	2.82	1.51
Close friends (*n* = 97)	7	7.50	21	22.94	0.89	1.24	3.02	1.26
***How often did you see him/her in school? ^+^***								
Never (*n* = 938)	59	6.27	68	7.31	0.57	1.02	2.98	1.45
Less than once a week (*n* = 619)	27	4.43	59	9.55	0.94	1.08	2.67	1.32
Once to four times a week (*n* = 431)	35	8.10	39	9.11	0.88	1.18	3.20	1.38
Daily (*n* = 863)	90	10.39	107	12.49	0.80	1.03	3.04	1.42
***How often did you see him or her after school or talk on the phone? ^+^***								
Never (*n* = 2167)	135	6.24	174	8.11	0.72	1.03	2.96	1.41
Less than once a week (*n* = 437)	49	11.22	61	13.92	1.01	1.20	2.87	1.53
Once to four times a week (*n* = 181)	17	9.24	30	16.76	0.66	0.99	3.00	1.27
Daily (*n* = 79)	10	12.17	9	12.85	1.13	1.38	3.05	1.49
***To what extent did you confide in each other? ^+^***								
Not at all (*n* = 2402)	151	6.29	194	8.15	0.72	1.04	2.95	1.42
Somewhat (*n* = 313)	49	15.69	57	18.14	0.97	1.18	2.89	1.36
Very much (*n* = 99)	8	8.45	16	16.82	0.87	1.31	3.20	1.44

^+^ 6 exposed schools (*n* = 2865); ^++^ 4 exposed schools (*n* = 1970); ^a^ Ns and percentages for each row; ^b^ Means and standard deviations for each row. The Friendship variables were assessed in stage 2; therefore, all results are weighted to adjust for unequal selection probabilities of stage 1 positives and negatives for inclusion in stage 2.

**Table 4:** Mixed effect regression analyses of serious suicidal ideation/behavior, depression, and attitudes about coping and help-seeking strategies by degree of friendship with suicide decedent.

Friendship Items	Serious Suicidal Ideation/Behavior		Depression		Maladaptive Coping Strategies		Adaptive Attitudes toward Help-Seeking	
	OR (95% CI)	*p*	OR (95% CI)	*p*	B (95% CI)	*p*	B (95% CI)	*p*
**Friendship scale**	1.11 (1.05–1.16)	<0.0001	1.14 ^+^ (1.09–1.20)	<0.001	0.04 (0.01–0.07)	0.022	0.02 (−0.02–0.05)	0.399
***How well did you know him/her?***								
-Not at all (ref)	-	-	-		-	-	-	-
-Acquaintances	1.11 (0.77–1.59)	0.581	1.44 (1.04–1.99)	0.0030	0.37 (0.18–0.57)	0.0002	0.10 (−0.16–0.36)	0.448
-Friends, but not close friends	1.85 (1.19–2.87)	0.006	2.20 (1.45–3.33)	0.0002	0.12 (−0.13–0.37)	0.354	−0.10 (−0.42–0.23)	0.558
-Close friends	1.12 (0.49–2.54)	0.793	4.40 (2.49–7.79)	<0.0001	0.20 (−0.31–0.71)	0.431	0.02 (−0.66–0.69)	0.958
***How often did you see him/her in school?***	1.29 (1.12–1.49)	0.0004	1.23 (1.08–1.39)	0.0017	0.09 (0.01–0.17)	0.020	0.06 (−0.03–0.14)	0.208
***How often did you see him or her after school or talk on the phone?***	1.29 (1.08–1.55)	0.0061	1.46 (1.24–1.72)	<0.0001	0.07 (−0.05–0.18)	0.259	0.02 (−0.14–0.17)	0.827
***To what extent did you confide in each other?***								
-Not at all (ref)	-	-	-	-	-	-	-	-
-Somewhat	2.68 (1.81–3.97)	<0.0001	2.63 (1.81–3.81)	<0.0001	0.22 (−0.05–0.49)	0.112	−0.01 (−0.36–0.34)	0.960
-A lot	1.20 (0.56–2.57)	0.643	2.24 (1.23–4.08)	0.0086	0.08 (−0.37–0.54)	0.722	0.25 (−0.35–0.85)	0.415

^+^ School is included as a fixed effect in the analyses in order for the model to converge. The Friendship variables were assessed in stage 2; therefore, all results are weighted to adjust for unequal selection probabilities of stage 1 positives and negatives for inclusion in stage 2.
